# Effects of Polymorphisms in *APOA4-APOA5-ZNF259-BUD13* Gene Cluster on Plasma Levels of Triglycerides and Risk of Coronary Heart Disease in a Chinese Han Population

**DOI:** 10.1371/journal.pone.0138652

**Published:** 2015-09-23

**Authors:** Qianxi Fu, Xiaojun Tang, Juan Chen, Li Su, Mingjun Zhang, Long Wang, Jinjin Jing, Li Zhou

**Affiliations:** 1 Department of Epidemiology, School of Public Health and Management, Chongqing Medical University, Chongqing, 400016, China; 2 The Second Affiliated Hospital and the Key Laboratory of Molecular Biology of Infectious Diseases designated by the Chinese Ministry of Education, Chongqing Medical University, Chongqing, 400016, China; 3 Department of Cardiology, the Second Affiliated Hospital of Chongqing Medical University, Chongqing, 400010, China; 4 Molecular Medicine and Tumor Research Center, Chongqing Medical University, Yuzhong District in Chongqing, 400016, China; University of Milan, ITALY

## Abstract

**Background/Aim:**

Recent genome-wide association studies have identified several loci influencing lipid levels. The present study focused on the triglycerides (TG)-associated locus, the *APOA4-APOA5-ZNF259-BUD13* gene cluster on chromosome 11, to explore the role of genetic variants in this gene cluster in the development of increasing TG levels and coronary heart disease (CHD).

**Methodology/Principal Findings:**

Six single nucleotide polymorphisms (SNPs), rs4417316, rs651821, rs6589566, rs7396835, rs964184 and rs17119975, in the *APOA4-APOA5-ZNF259-BUD13* gene cluster were selected and genotyped in 5374 healthy Chinese subjects. There were strong significant associations between the six SNPs and TG levels (*P*<1.0×10^−8^). Moreover, a weighted genotype score was found to be associated with TG levels (*P* = 3.28×10^−13^). The frequencies of three common haplotypes were observed to be significantly different between the high TG group and the low TG group (*P*<0.05). However, no significant effects were found for the SNPs regarding susceptibility to CHD in the Chinese case-control populations.

**Conclusions/Significance:**

This study highlights the genotypes, genotype scores and haplotypes of the *APOA4-APOA5-ZNF259-BUD13* gene cluster that were associated with TG levels in a Chinese population; however, the genetic variants in this gene cluster did not increase the risk of CHD in the Chinese population.

## Introduction

Plasma levels of triglycerides (TG) are associated with a future risk of cardiovascular disease [1, 2]. Recent genome-wide association studies (GWASs) have identified common variants that contribute to plasma lipid levels including TG [3–5]. Willer et al. performed the joint GWAS and Metabochip meta-analysis, which identified 39 loci that were associated with TG, and found that these loci were associated with cardiovascular and metabolic traits including coronary heart disease (CHD) [6]. We also conducted a GWAS to identify the variants associated with lipid levels in a Chinese Han population of 12,281 individuals. In that study, we replicated two genetic loci that were strongly associated with TG levels. The two loci were the *LPL* gene on chromosome 8 and the *APOA4-APOA5-ZNF259-BUD13* gene locus on chromosome 11. The polymorphism rs651821 in the *APOA4-APOA5-ZNF259-BUD13* gene locus had the strongest association with TG levels in the Han Chinese population (combined *P* = 2.38×10^−59^) [7]. A large GWAS for CHD reported that another single nucleotide polymorphism (SNP), rs964184 in the *APOA4-APOA5-ZNF259-BUD13* gene cluster, was also associated with the presence of CHD in subjects with European or south Asian descent [8, 9]. However, there was a lack of evidence concerning the effect of SNPs in the *APOA4-APOA5-ZNF259-BUD13* gene cluster on cardiovascular risk, particularly in a Chinese Han population.

In the present study, we focused on the *APOA4-APOA5-ZNF259-BUD13* gene locus and investigated six variants. The six variants are located in an approximately 55 kb region surrounding the two SNPs rs651821 and rs964184, which were significantly associated with lipid levels and CHD risk in previous studies. The polymorphisms are rs17119975, rs964184, rs4417316, rs6589566, rs651821 and rs7396835. The aim of this study was to investigate the effects of genotype scores and haplotypes based on the variants in the *APOA4-APOA5-ZNF259-BUD13* gene cluster on TG levels. In addition, we examined the association between the SNPs and cardiovascular risk in a Chinese Han case-control population.

## Materials and Methods

### Subjects

We performed two independent study populations. A total of 5347 subjects from the general population were used in the present study; they were healthy subjects who were recruited during a routine health examination and who had no diagnosed chronic diseases (Table 1). The CHD case-control study population, including 1376 CHD patients and 1376 age- and sex-frequency matched healthy control subjects, was described in a previous study [10, 11]. The inclusion criteria for the CHD case subjects were stenoses ≥ 50% in at least 1 major coronary artery, which was identified by coronary angiography and/or a diagnosis of CHD based on the World Health Organization criteria. The control subjects, who were residing in the same communities as the cases, were determined to be free of CHD and peripheral atherosclerotic arterial disease by medical history, clinical examinations and electrocardiography. The criteria proposed for a clinical diagnosis of elevated TG levels was that developed by the NCEP ATP III arbitrarily: TG levels less than 1.7 mmol/L were normal; TG levels greater than 1.7 mmol/L were elevated [12]. The clinical threshold of 1.7 mmol/L for TG levels was used to divide the subjects into ‘low’ or ‘high’ TG groups in our study. Structured questionnaires were used by trained interviewers to collect information on age, sex, smoking and drinking. Smoking and drinking behaviors were assessed on the basis of a self-administered lifestyle questionnaire. The subjects were classified as smokers and nonsmokers. Those who had smoked less than 100 cigarettes in their lifetime were defined as nonsmokers; otherwise, they were defined as smokers. Alcohol consumption was classified into two categories: drinkers and nondrinkers. Respondents that reported drinking any alcoholic beverage ‘less than once a year’ or ‘never’ were coded as nondrinkers; otherwise, they were defined as drinkers. Body mass index (BMI) was calculated as (weight [kg]/height [meter]^2^). The subjects were all of self-reported Chinese Han ethnicity. All the experiments were performed in accordance with relevant guidelines and regulations. All participants signed a written informed consent for the investigations. The Ethical Committee of Chongqing Medical University approved the present study.

**Table 1:** General characteristics of the study participants in the Chinese Han population.

		Participants	TG (mmol/L)	*P*
Age	mean (S.D.)	62.8 (8.2)		
	≤60	1781 (41.38%)	1.47±1.00	0.25
	>60	2523 (58.62%)	1.51±1.03	
Sex	Male	1846 (42.45%)	1.46±1.05	0.08
	Female	2503 (57.55%)	1.52±1.04	
Body mass index (kg/m^2^)	<25	2476 (60.10%)	1.31±0.08	<0.001
	≥25	1644 (39.90%)	1.77±1.21	
Smoking	Yes	1148 (26.78%)	1.47±0.98	0.63
	No	3182 (73.22%)	1.49±1.03	
Drinking	Yes	1076 (24.73%)	1.44±0.97	0.07
	No	3274 (75.27%)	1.50±1.04	

Data are the mean values and SD for quantitative traits (age and TG levels) and absolute counts and percentages for binary traits.

### SNP selection

A total of six SNPs were genotyped in our study, including 4 haplotype-tagging SNPs and two previously reported susceptibility SNPs (rs651821 and rs964184) [4, 7]. These SNPs cover a 55 kb genomic region on chromosome 11 (the National Centre for Biotechnology Information build GRCh36 from 116135000 to 116190000). The initial set of 19 common variants (minor allele frequency >0.1) in this region can be well captured (r^2^ = 0.8) by this set of 4 haplotype-tagging SNPs, which were selected from the genotyped SNPs in the Chinese Han population of the HapMap project (the PhaseII database) using the pairwise tagging method in Haploview4.0 [13].

### Genotyping

Blood samples were collected from each individual in ethylenediaminetetraacetic (EDTA) tubes, and genomic DNA was isolated using a Tiangen DP319-02 kit (Tiangen Company, China). Genotyping was performed according to the Mass Array time-of-flight mass spectrometer (Sequenom Company, USA). Polymerase chain reaction and extension primers were designed using Mass ARRAY Assay Design 3.1 software (Sequenom Company, USA). The genotyping procedures were performed according to the manufacturer’s iPLEX Application Guide (Sequenom Company, USA). All the genotyping reactions were performed in 384-well plates. Each plate included four randomly selected duplicates and six negative controls using double distilled water. The average concordance rate for the genotypes was 99.5%.

### Statistical analysis

The analysis for the TGs was log-transformed. The effects of the genotypes on plasma lipid levels were assessed by multiple linear regression models with adjustment for age, sex and BMI. The association between SNPs and CHD risk was estimated by computing odds ratios (ORs) and 95% confidence intervals (CIs) from the multivariate logistic regression analyses. The Bonferroni correction method was applied to correct for multiple testing.

The genotype score was calculated on the basis of five SNPs that were tagging lipid-associated genes in 5374 healthy Chinese subjects. A weighted genotype score was calculated by multiplying the number of risk alleles in each SNP (0, 1 or 2) for the corresponding beta coefficient from multiple linear regression models and then taking the sum of the five SNPs. The effects of the genotype scores on lipid levels were also assessed by multiple linear regression models with adjustment for age, sex and BMI.

A haplotype analysis of five SNPs in the *APOA4-APOA5-ZNF259-BUD13* gene cluster was performed by a Phase 2.0 program [14]. A chi-square test was used to compare the frequency of haplotypes between low TG group (TG levels <1.70 mmol/L) and the high TG group (TG levels ≥1.70 mmol/L). Haplotypes with frequencies of less than 0.01 were excluded from the analysis. The probability level accepted for significance was *P*<0.05. The statistical analyses were performed using SPSS 17.0 software (SPSS Inc., Chicago, Illinois) if not stated otherwise.

## Results

**Fig 1:**
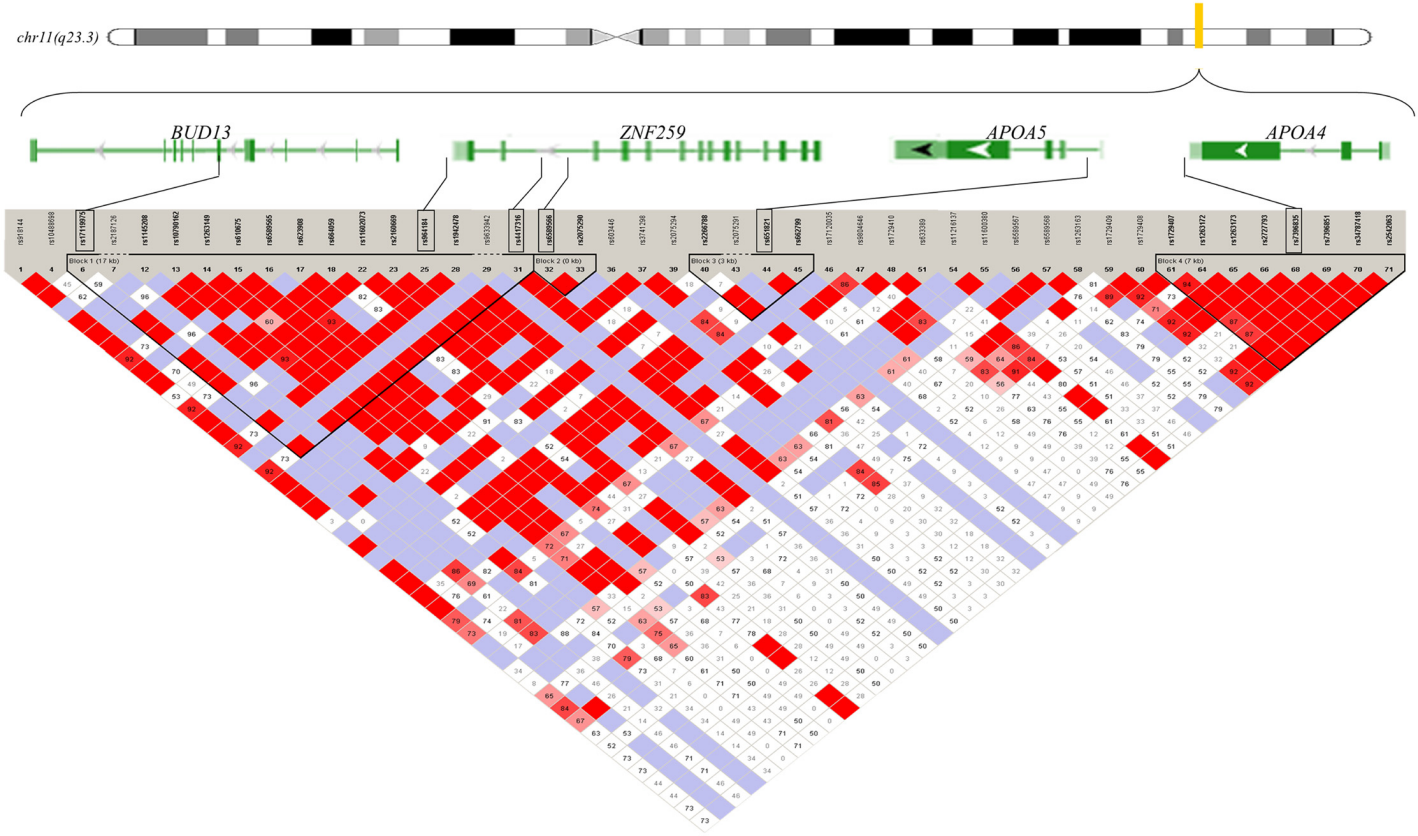
Linkage disequilibrium patterns between genotyped SNPs around the *APOA4-APOA5-ZNF259-BUD13* gene cluster in a Chinese Han population. The location of the genetic markers in the chromosome region (11q23.3) (55 kb) encompasses *APOA4*, *APOA5*, *ZNF259* and *BUD13*. The direction of transcription of the genes is shown in arrows. The pair-wise LD between the SNPs is indicated by diamonds shaded in white-gray-red, which show the range of the LD matrix from D’ = 0 in white to D’ = 1 in red. The position of the six SNPs, rs17119975, rs964184, rs4417316, rs6589566, rs651821 and rs7396835, on the genes is indicated by the black rectangles.

The general characteristics and TG levels of the participants are presented in Table 1. In the 5347 subjects, BMI was significantly associated with TG levels (*P*<0.001). The TG levels were significantly higher in subjects whose BMI ≥25 kg/m^2^. However, no associations were observed between age, sex, drinking, smoking and TG levels. The linkage disequilibrium (LD) pattern between the six SNPs in the *APOA4-APOA5-ZNF259-BUD13* gene cluster in the Chinese Han population is shown in Fig 1 and S1 Table.

We investigated the association between the six SNPs and lipid TG levels in 5347 healthy Chinese participants. The six SNPs were all in the *APOA4-APOA5-ZNF259-BUD13* gene cluster: rs17119975 is near *BUD13*; rs964184, rs4417316 and rs6589566 are near *ZNF259*; rs651821 is near *APOA5*; and rs7396835 is near *APOA4*. Details of the investigated SNPs, chromosomal positions, nearby genes, genotypes, allele frequencies and effect sizes are summarized in Table 2. There was a significant correlation between the genotypes of the six SNPs and TG levels (all *P* values <0.008 after Bonferroni corrections). The best overall signal was observed for SNP rs651821 (*P* = 2.35×10^−32^, Table 2). In the six SNPs, rs964184 was highly correlated (r^2^ = 0.885, S1 Table) with rs651821. We did conditional analysis to assess which SNP was independently associated with the TG levels. The results showed that no SNP remained significant *P*<0.008 after analyses conditioning on rs651821.

**Table 2:** Associations between polymorphisms in the *APOA4-APOA5-ZNF259-BUD13* gene cluster and TG levels in 5347 Chinese Han participants.

SNP	Position ^a^	Near gene	Genotype (major/minor)	MAF ^b^	Effect size (s.e.m.)^c^	*P* ^d^
rs17119975	116139767	*BUD13*	T/C	0.232	-0.089 (0.017)	1.79×10^−9^
rs964184	116154127	*ZNF259*	C/G	0.226	0.117 (0.024)	9.28×10^−18^
rs4417316	116157511	*ZNF259*	C/T	0.280	-0.107 (0.022)	3.34×10^−13^
rs6589566	116157633	*ZNF259*	A/G	0.226	0.125 (0.019)	1.96×10^−18^
rs651821	116167789	*APOA5*	T/C	0.262	0.170 (0.014)	2.35×10^−32^
rs7396835	116189238	*APOA4*	C/T	0.339	0.100 (0.020)	3.86×10^−12^

^a^Chromosomal positions are based on the NCBI Build 36 of the genome.

^b^MAF, minor allele frequency.

^c^The minor allele is the effect allele, and the major allele is the reference allele.

^d^The *P* values for the effects of genotypes on plasma TG levels were assessed by multiple linear regression models with adjustment for age, sex and BMI.

To determine whether the six SNPs in the *APOA4-APOA5-ZNF259-BUD13* gene cluster account for any other associations with TG levels when tested together, we performed a haplotype analysis. The subjects whose TG levels were greater than 1.70 mmol/L were classified as the high TG group; the remaining subjects were in the low TG group. Given that 2 SNPs (rs964184 and rs6589566) were in full LD (D’ = 1.0, r^2^ = 1.0, based on the HapMap project Phase II database), rs6589566 can encompass rs964184 (S1 Table). Therefore, the five risk SNPs (rs17119975, rs4417316, rs6589566, rs651821 and rs7396835) were selected for further analysis. There were 25 haplotypes in the high TG group and 28 haplotypes in the low TG group. The haplotypes with frequencies less than 1% were excluded from further analyses. Finally, we compared seven haplotypes between the high TG group and the low TG group. As shown in Table 3, compared with the haplotype CTATC, which included lower TG related alleles in each SNP, three other haplotypes (TCGCT, TCGCC and TCACC) had significantly different frequencies (*P*<0.05). The less frequent haplotype, TCACC, had the strongest association with increasing TG levels (OR = 2.22, 95% CI = 1.57–3.14, *P* = 4.91×10^−6^).

**Table 3:** Associations of the haplotypes derived from the five polymorphisms in 5347 Chinese participants.

Polymorphisms ^a^					Haplotype frequencies ^b^			
rs17119975 (*BUD13*)	rs4417316 (*ZNF259*)	rs6589566 (*ZNF259*)	rs651821 (*APOA5*)	rs7396835 (*APOA4*)	Low TG group (<1.70 mmol/L) N = 3066(%)	High TG group (≥1.70 mmol/L) N = 1199(%)	*P*	OR(95% CI)
C	T	A	T	C	632(20.62)	190(15.83)	-	1.00(Reference)
T	C	A	T	C	1068(34.84)	387(31.58)	0.07	1.21(0.99–1.47)
T	C	G	C	T	389(12.68)	218(18.17)	1.87×10^−7^	1.86(1.48–2.35)
T	C	A	T	T	313(10.2)	117(9.74)	0.11	1.24(0.95–1.62)
T	C	G	C	C	212(6.91)	97(8.09)	4.00×10^−3^	1.52(1.11–2.03)
T	C	A	C	C	102(3.33)	68(5.71)	4.91×10^−6^	2.22(1.57–3.14)
C	T	A	T	T	157(5.12)	55(4.62)	0.39	1.17(0.82–1.65)

^a^The reference haplotype CTATC included fewer TG related alleles in each SNP.

^b^The criteria proposed for the clinical diagnosis of elevated TG levels by the NCEP ATP III arbitrarily: the clinical threshold of 1.7 mmol/L for TG levels was used to divide the subjects into ‘low’ or ‘high’ TG groups in our study.

Chi-square tests were used to compare the frequency of haplotypes between the low TG group and the high TG group.

**Fig 2:**
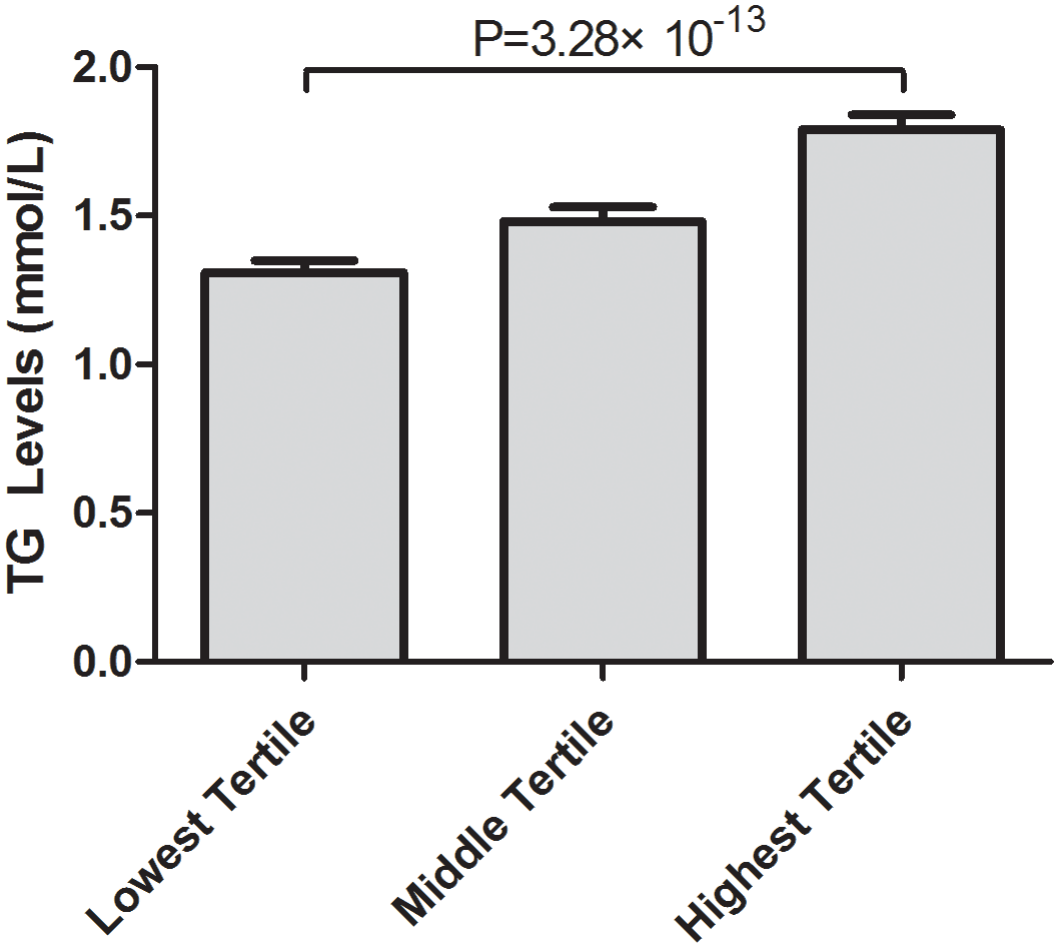
Association between tertiles of weighted genotype scores comprising five risk SNPs with TG levels. The tertiles of the weighted genotype scores comprised five risk SNPs (rs17119975, rs4417316, rs6589566, rs651821 and rs7396835) and TG levels. Lowest tertile (n = 1677), middle tertile (n = 2135), highest tertile (n = 1368).

We next tested whether the cumulative allelic dosage of risk alleles in the five SNPs contributed to the quantitative variation in the TG levels observed in the 5347 subjects in this Chinese population. As shown in Fig 2, we divided the general population into tertiles by weighted genotype score and found a significant association between the tertiles of cumulative risk score and stepwise increased TG levels (*P* = 3.28×10^−13^), indicating that there was a significant dose-response relationship between the risk allele and TG levels in the *APOA4-APOA5-ZNF259-BUD13* gene cluster.

Furthermore, we investigated the association between the five TG-associated SNPs in the *APOA4-APOA5-ZNF259-BUD13* gene cluster and the risk of CHD. As shown in Table 4, the frequencies of the three genotypes in each TG-associated SNP were similar in the CHD cases and controls. These results indicated that there was no significant association between the six TG-associated SNPs and CHD risk in this Chinese population (all *P* > 0.05).

**Table 4:** Associations of the five TG-associated SNPs on the *APOA4-APOA5-ZNF259-BUD13* gene cluster with CHD risk in a Chinese Han population.

	Genotype	CHD cases, n (%)	Controls, n (%)	Adjusted^a^ OR (95% CI)	Adjusted ^a^ *P*
rs17119975					
	TT	371 (60.0)	393 (59.2)	1.00	
	TC	259 (38.3)	258 (38.9)	1.06 (0.85–1.33)	0.59
	CC	10 (1.7)	13 (1.9)	0.82 (0.47–1.88)	0.63
rs4417316					
	CC	345 (55.8)	366 (55.1)	1.00	
	CT	228 (36.9)	245 (37.0)	0.99 (0.78–1.25)	0.91
	TT	45 (7.3)	52 (7.9)	0.92 (0.60–1.41)	0.69
rs6589566					
	AA	329 (53.3)	355 (53.5)	1.00	
	AG	272 (44.1)	291 (43.8)	1.01 (0.81–1.26)	0.94
	GG	16 (2.6)	18 (2.7)	0.96 (0.48–1.91)	0.91
rs651821					
	*TT*	702(53.6)	683(53.2)	1.00	
	*TC*	501(38.3)	492(38.3)	1.02(0.85–1.21)	0.87
	*CC*	106(8.1)	110(8.5)	1.13(0.88–1.35)	0.44
rs7396835					
	CC	172 (27.9)	186 (28.1)	1.00	
	CT	379 (61.4)	406 (61.1)	1.01 (0.79–1.30)	0.94
	TT	66 (10.7)	72 (10.8)	0.99 (0.67–1.47)	0.96

^a^Data were calculated by a logistic regression analysis with adjustment for age, sex, smoking and BMI.

## Discussion

The present study has convincingly replicated the association of the six SNPs in the gene cluster that is the most associated with plasma TG levels in a Chinese Han population. We previously reported eight loci that were associated with lipid levels in Chinese subjects. Among these lipid-associated loci, the SNP rs651821 in the *APOA4-APOA5-ZNF259-BUD13* gene cluster had the strongest association with TG levels in a previous GWAS in Chinese subjects [7]. Our current data also provide strong evidence of the association of rs651821 and five other SNPs in this gene cluster with TG levels in the current Chinese population of 5347 individuals. In addition, our results suggested that the cumulative effect of multiple genetic variants contributed to TG levels. However, we did not observe a relationship between the genetic variants and CHD risk. These results suggest that this strongly associated region should be sequenced fully and investigated for true functional variants and potential clinical applications.

The *APOA4-APOA5-ZNF259-BUD13* gene cluster on chromosome 11 has been reported to be associated with increased TG levels in various studies [3–7, 15, 16]. Among the six SNPs, rs964184 and rs651821 were previously reported to be significantly associated with TG levels in European and Chinese Han populations [4, 7]. We also successfully observed a significant association of this locus with higher TG levels in the present study. Upon analyzing these variants together in the haplotype analysis, compared with the haplotype CTATC, which carried fewer TG related alleles in each SNP, three other haplotypes (TCGCT, TCGCC and TCACC) revealed strong associations with higher TG levels. The haplotypes containing risk alleles for two SNPs (the G allele of rs6589566 and the C allele of rs651821) were significantly associated, whereas the haplotypes with non-risk alleles for these markers showed no evidence of association. This discovery indicated that the major effect appeared to be driven by two SNPs rs6589566 and rs651821. Interestingly, rs6589566 in *ZNF259* and rs651821 in *APOA5* had the strongest association with TG levels in the single marker analyses in the present study. rs6589566 was also in full LD with rs964184 in this Chinese Han population; therefore, it could encompass rs964184. Meanwhile, the most significantly TG-associated SNP rs651821 was highly correlated with the two SNPs rs6589566 and rs964184 (r^2^ = 0.885). Our conditional analyses refined the top SNP rs651821 was independently associated with the TG levels, which was consistent with the previous studies [7, 16].

We also found some evidence of a cumulative effect in common risk alleles. There was a strong association between TG levels and an increasing genotype score of risk alleles. These findings indicated that the functional properties of multiple variants might act together to influence the development of disease. The genetic profiles such as the genotype score and haplotype may be useful for the early detection and treatment of dyslipidemias, thus enabling early preventive strategies [17, 18].

The *APOA4-APOA5-ZNF259-BUD13* gene cluster locus in the chromosome region 11q23.3 is approximately a 55 kb region. This region includes lipoprotein encoding genes such as *APOA4* and *APOA5*. However, the exact function of the other two genes *ZNF259* and *BUD13* in lipid mechanisms was unclear. *ZNF259* encodes a regulatory protein that is involved in cell proliferation and signal transduction [19]. *BUD13* is one of the subunits of the splicing factor that affects nuclear pre-mRNA retention [20]. Many GWASs have reported that the G allele of rs964184, which resides in the intergenic region between *ZNF259* and *BUD13*, was strongly associated with increased TG levels. Aung et al. also noted that rs964184 was associated with TG levels in two different Chinese populations and displayed ethnic or sex specificity [21, 22]. The six SNPs in this gene cluster belong to intergenic or noncoding regions. These SNPs may influence the transcriptional binding sites of the adjacent genes or contribute to transcriptional mechanisms without being directly involved in protein regulation. Therefore, further study requires using deep sequencing or functional experiments to clarify which SNPs are causal and how they actually influence circulating TG levels.

This locus included obvious functional candidate genes such as *APOA5* and was known to be associated with cardiovascular outcomes. Cui et al. recently reported that rs2266788, which is located in the 3’UTR of *APOA5*, contributed to elevated TG levels and the severity of CHD by interfering with microRNA 3201 binding efficiency [23]. We tested the association between the five TG-related SNPs and the risk of CHD in a large Chinese case-control population. However, we observed no association between the variants and susceptibility to CHD. Woestijne et al. also reported that another SNP in this region, rs964184, was not related to vascular events in Europeans [24], which was consistent with our results. Increasing evidence has shown that high TG levels and genetic variation are associated with increased cardiovascular morbidity and mortality risk [25]. However, genetic variation explains only 5–50% of overall TG variation and CHD risk [4, 17], suggesting that many determinants have yet to be clarified. Lifestyle and environmental variation may affect observed associations between the genetic variants and disease outcomes. Although genotype cannot be changed, the phenotype may be influenced by increasing body weight, and our results underscore the importance of advocating weight loss for overweight subjects with high TG levels.

There are several limitations of the present study that must be acknowledged. In comparisons of genome sequences in Southern and Northern Han Chinese populations, differences in several disease phenotypes have revealed significant variation between Han Chinese populations [26]. Hence, the subjects recruited in our study may not be entirely representative of the general Han Chinese population. Second, there may have been some selection bias in our CHD case-control study (the inclusion of surviving CHD patients). The controls in the present study did not undergo coronary angiography, and some of the controls may have had undiagnosed CHD, which might bias our results toward the null. Third, the sample size in this study was relatively low compared to many GWASs. Further prospective cohort studies with larger sample sizes are required to confirm our results.

In conclusion, we have replicated the strong associations of six SNPs in the *APOA4-APOA5-ZNF259-BUD13* gene cluster with TG levels in a Chinese Han population. Haplotype and genotype score analyses also demonstrated the genetic cumulative effect on TG levels in this region. However, we did not observe an association between the SNPs in this gene cluster and CHD risk. Further studies are required to investigate potential mechanisms underlying the links between the genetic variants in the *APOA4-APOA5-ZNF259-BUD13* gene cluster, TG levels and CHD risk.

## Supporting Information

S1 Table The details of the Linkage disequilibrium and r^2^ among the six SNPs in APOA4-APOA5-ZNF259-BUD13 gene cluster in Chinese Han population. (DOC)

S2 Table Results of the stratification concerning traditional risk factors such as age, sex, BMI and smoking for five SNPs and TG levels. (DOC)
