# Supplementary material for: Effects of Polymorphisms in APOA4-APOA5-ZNF259-BUD13 Gene Cluster on Plasma Levels of Triglycerides and Risk of Coronary Heart Disease in a Chinese Han Population
Source: PLoS One. 2015 Sep 23;10(9):e0138652. doi: 10.1371/journal.pone.0138652 (PMC4580433; doi:10.1371/journal.pone.0138652)
Supplement: S1 Table — (DOC) [file pone.0138652.s001.doc]

**S1 Table. The details of the Linkage disequilibrium and r^2^ among the six SNPs in *APOA4-APOA5-ZNF259-BUD13* gene cluster in Chinese Han population.**

| SNP | r^2^ (D’) | | | | | |
| --- | --- | --- | --- | --- | --- | --- |
|  | SNP | | | | | |
|  | rs17119975 | rs964184 | rs4417316 | rs6589566 | rs651821 | rs7396835 |
| rs17119975 | — | 0.086(1.0) | 0.684(1.0) | 0.086(1.0) | 0.097(1.0) | 0.091(0.71) |
| rs964184 | 0.086(1.0) | — | 0.125(1.0) | 1.000(1.0) | 0.885(1.0) | 0.129(0.524) |
| rs4417316 | 0.684(1.0) | 0.125(1.0) | — | 0.125(1.0) | 0.141(1.0) | 0.065(0.494) |
| rs6589566 | 0.086(1.0) | 1.000(1.0) | 0.125(1.0) | — | 0.885(1.0) | 0.129(0.524) |
| rs651821 | 0.097(1.0) | 0.885(1.0) | 0.141(1.0) | 0.885(1.0) | — | 0.121(0.477) |
| rs7396835 | 0.091(0.710 | 0.129(0.524) | 0.065(0.494) | 0.129(0.524) | 0.121(0.477) | — |
