# Supplementary material for: Effects of Polymorphisms in APOA4-APOA5-ZNF259-BUD13 Gene Cluster on Plasma Levels of Triglycerides and Risk of Coronary Heart Disease in a Chinese Han Population
Source: PLoS One. 2015 Sep 23;10(9):e0138652. doi: 10.1371/journal.pone.0138652 (PMC4580433; doi:10.1371/journal.pone.0138652)
Supplement: S2 Table — (DOC) [file pone.0138652.s002.doc]

**S2 Table. Results of the stratification concerning traditional risk factors such as age, sex, BMI and smoking for five SNPs and TG levels.**

| SNP | Genotype | TG (mmol/L) | | | | | | | | | | | | | |
| --- | --- | --- | --- | --- | --- | --- | --- | --- | --- | --- | --- | --- | --- | --- | --- |
|  |  | Gender | |  | Smoking | |  | Drinking | |  | Age | |  | BMI (kg/m^2^) | |
|  |  | Male | Female |  | Yes | No |  | Yes | No |  | <60 | ≥60 |  | <25 | ≥25 |
| rs17119975 | CC | 1.15±0.56 | 1.40±0.88 |  | 1.13±0.48 | 1.35±0.85 |  | 1.20±0.59 | 1.33±0.83 |  | 1.26±0.78 | 1.30±0.77 |  | 1.16±0.64 | 1.49±0.88 |
|  | CT | 1,37±0.89 | 1.44±0.99 |  | 1.44±1.02* | 1.40±0.93* |  | 1.39±0.98 | 1.42±0.95 |  | 1.41±1.09 | 1.42±0.84 |  | 1.29±0.81 | 1.63±1.12 |
|  | TT | 1.55±1.16* | 1.58±1.03* |  | 1.54±1.00 | 1.57±1.12* |  | 1.51±1.00* | 1.59±1.11* |  | 1.53±0.97* | 1.59±1.15* |  | 1.35±0.86* | 1.89±1.29* |
|  | *P* | 1.34×10^-5^ | 0.002 |  | 0.001 | 1.52×10^-5^ |  | 0.017 | 5.05×10^-6^ |  | 0.003 | 1.43×10^-5^ |  | 0.006 | 1.54×10^-5^ |
| rs4417316 | TT | 1.13±0.56 | 1.34±0.84 |  | 1.11±0.47 | 1.32±0.81 |  | 1.15±0.61 | 1.30±0.78 |  | 1.23±0.75 | 1.26±0.73 |  | 1.15±0.63 | 1.44±0.83 |
|  | CT | 1.40±0.95* | 1.44±0.99* |  | 1.47±1.10* | 1.39±0.92 |  | 1.43±1.00* | 1.42±0.96 |  | 1.41±1.06 | 1.43±0.90 |  | 1.28±0.79* | 1.64±1.16* |
|  | CC | 1.56±1.16* | 1.59±1.03* |  | 1.53±0.94* | 1.59±1.14* |  | 1.50±0.99* | 1.61±1.11* |  | 1.56±0.99* | 1.60±1.14* |  | 1.36±0.88 | 1.91±1.28 |
|  | *P* | 3.23×10^-6^ | 7.76×10^-5^ |  | 4.78×10^-4^ | 4.13×10^-8^ |  | 0.006 | 2.40×10^-8^ |  | 1.85×10^-4^ | 1.40×10^-6^ |  | 3.14×10^-4^ | 1.65×10^-6^ |
| rs651821 | CC | 1.30±0.78 | 1.36±0.77 |  | 1.35±0.80 | 1.33±0.76 |  | 1.30±0.80 | 1.35±0.77 |  | 1.33±0.76 | 1.35±0.78 |  | 1.21±0.70 | 1.54±0.83 |
|  | CT | 1.55±1.18* | 1.64±1.18* |  | 1.58±1.08* | 1.61±1.21* |  | 1.53±1.02* | 1.63±1.22* |  | 1.59±1.18* | 1.62±1.19* |  | 1.38±0.88* | 1.98±1.48* |
|  | TT | 1.95±1.37* | 1.90±1.29* |  | 1.73±1.08* | 1.97±1.40* |  | 1.87±1.27* | 1.93±1.33* |  | 1.89±1.30* | 1.92±1.28* |  | 1.68±1.16* | 2.24±1.43* |
|  | *P* | 1.03×10^-13^ | 4.36×10^-17^ |  | 3.39×10^-5^ | 2.64×10^-25^ |  | 4.46×10^-8^ | 5.49×10^-22^ |  | 3.06×10^-12^ | 6.85×10^-17^ |  | 1.13×10^-14^ | 1.48×10^-16^ |
| rs6589566 | AA | 1.36±0.88 | 1.42±0.93 |  | 1.40±0.86 | 1.39±0.92 |  | 1.34±0.86 | 1.42±0.92 |  | 1.38±0.93 | 1.40±0.89 |  | 1.25±0.78 | 1.61±1.03 |
|  | AG | 1.60±1.25* | 1.64±1.08* |  | 1.60±1.11* | 1.64±1.17 |  | 1.57±1.05* | 1.65±1.18* |  | 1.60±1.09* | 1.64±1.18* |  | 1.39±0.91* | 2.01±1.41* |
|  | GG | 1.70±1.01* | 1.83±1.31* |  | 1.58±0.97* | 1.82±1.25* |  | 1.77±1.09* | 1.76±1.22* |  | 1.74±1.15 | 1.79±1.23 |  | 1.54±0.88 | 2.08±1.43 |
|  | *P* | 1.04×10^-6^ | 4.72×10^-9^ |  | 0.002 | 1.37×10^-12^ |  | 3.13×10^-5^ | 1.94×10^-10^ |  | 3.72×10^-6^ | 3.69×10^-9^ |  | 7.32×10^-6^ | 1.85×10^-10^ |
| rs7396835 | CC | 1.38±0.88 | 1.40±0.87 |  | 1.43±0.94 | 1.37±0.86 |  | 1.38±0.94 | 1.39±0.86 |  | 1.37±0.85 | 1.40±0.90 |  | 1.23±0.77 | 1.63±0.96 |
|  | CT | 1.54±1.23* | 1.59±1.06 |  | 1.53±1.07 | 1.58±1.15* |  | 1.49±1.02 | 1.60±1.17* |  | 1.54±1.09* | 1.58±1.16* |  | 1.37±0.87* | 1.89±1.41* |
|  | TT | 1.52±0.92* | 1.72±0.74* |  | 1.82±1.25 | 1.69±1.18* |  | 1.65±1.12 | 1.65±1.12* |  | 1.64±1.23* | 1.64±1.01* |  | 1.40±0.88* | 1.97±1.27* |
|  | *P* | 0.005 | 8.70×10^-8^ |  | 0.017 | 1.13×10^-9^ |  | 0.012 | 1.54×10^-8^ |  | 1.52×10^-4^ | 2.06×10^-6^ |  | 1.36×10^-4^ | 1.66×10^-5^ |

The *P* values for SNP effect calculated in a multiple linear regression model adjusted for age, sex, BMI, smoking and drinking.
